# Supplementary material for: Temperature and competition: drivers in the ecological dynamics of Aedes mosquitoes and dengue spread
Source: Parasit Vectors. 2026 Feb 22;19:137. doi: 10.1186/s13071-025-07187-y (PMC13037009; doi:10.1186/s13071-025-07187-y)
Supplement: Supplementary file 1 — (pdf 259 KB) [file 13071_2025_7187_MOESM1_ESM.pdf]

Supplementary information

Effects on dengue transmission

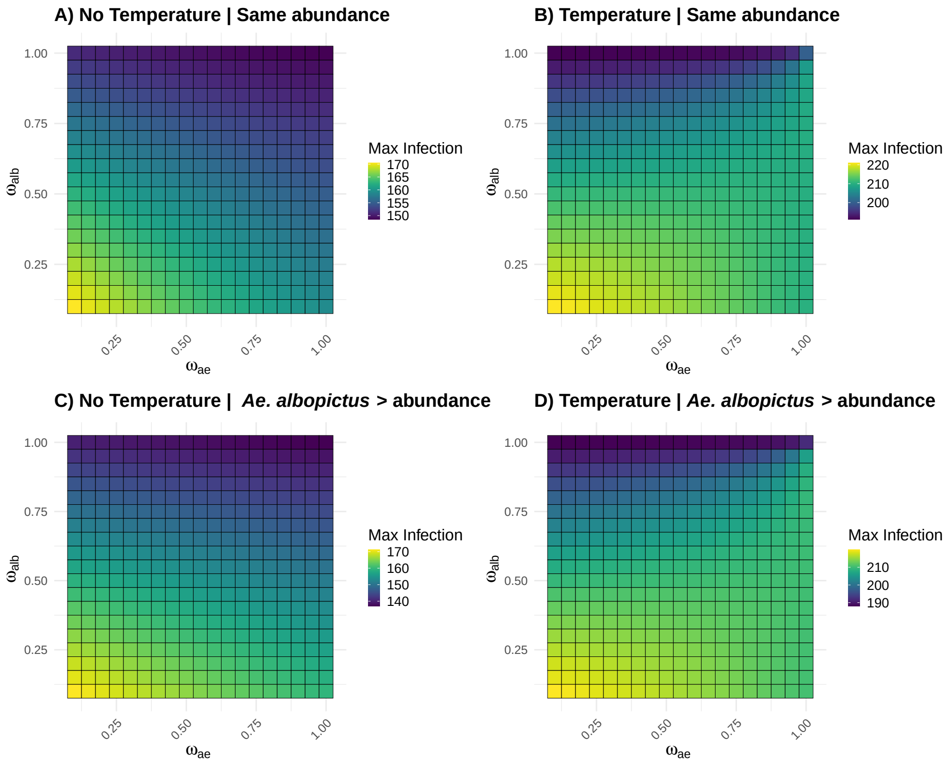

Additional file 1: Fig. S1. Dengue transmission dynamics under equal abundances and *Aedes albopictus* dominance scenarios. Heat maps comparing infection patterns across initial abundance compositions and temperature conditions. Panels A-B: Equal initial abundances showing infection values of 150-170 humans under temperature-independent conditions (Panel A) and 190-220 humans under temperature-dependent conditions (Panel B). Panels C-D: *Ae. albopictus* dominance scenarios displaying the lowest transmission potential with human case counts of 140-170 humans under temperature-independent conditions (Panel C) and 190-215 humans under temperature-dependent conditions (Panel D). Across all scenarios, peak infections consistently occur when both larval competition coefficients (ω_{ae} and ω_{alb}) approach minimal values near 0, while maximum *Ae. albopictus* competition coefficients (0.75-1.00) produce the lowest case counts. Temperature-dependent conditions consistently elevate transmission potential by 25-30% compared to their temperature-independent counterparts, regardless of initial abundance composition. Color scales represent the number of infected humans, with spatial patterns demonstrating the constraining effect of interspecific competition on dengue transmission dynamics.

Calculation of the Basic Reproductive Number (R_0)

Additional file 1: Text. S2. In this section, we detail the calculation of the basic reproductive number (R_0) for the mosquito-human competition model. The state variables and parameters are defined as follows:

State Variables

- S: Susceptible humans.
- E: Exposed humans.
- I: Infected humans.
- Y_{ae}: Infected *Aedes aegypti* mosquitoes.
- Y_{alb}: Infected *Aedes albopictus* mosquitoes.

Parameters

- N_h: Total human population.
- p_D: Probability of disease transmission per bite.
- BR_{ae}, BR_{alb}: Mosquito biting rates for *Aedes aegypti* and *Aedes albopictus*, respectively.
- ζ_{ae}, ζ_{alb}: Transmission efficiency from human to mosquito
- X_{ae}, X_{alb}: Mosquito population sizes for *Aedes aegypti* and *Aedes albopictus*.
- θ_h: Rate of progression from exposed to infected in humans.
- γ_h: Recovery rate of infected humans.
- μ_{Aae}, μ_{Aalb}: Mortality rates of infected *Aedes aegypti* and *Aedes albopictus*.

Next-Generation Matrix

The infection dynamics are governed by the following vectors of new infections ($\mathbf{F}$) and transitions ($\mathbf{V}$) (see subsection 5):

$$\mathbf{F} = \begin{pmatrix} \frac{p_D(BR_{ae}Y_{ae}+BR_{alb}Y_{alb})}{N_h}S \\ 0 \\ \frac{\zeta_{ae}X_{ae}I}{N_h}\left(1-\frac{Y_{ae}}{K_{ad}}\right) \\ \frac{\zeta_{alb}X_{alb}I}{N_h}\left(1-\frac{Y_{alb}}{K_{alb}}\right) \end{pmatrix},$$

$$\mathbf{V} = \begin{pmatrix} \theta_h E \\ -\theta_h E + \gamma_h I \\ \mu_{Aae}Y_{ae} \\ \mu_{Aalb}Y_{alb} \end{pmatrix}.$$

The Jacobian matrices of **F** and **V** evaluated at the disease-free equilibrium yield the next-generation matrix:

$$\mathcal{K} = \begin{bmatrix} 0 & 0 & \frac{BR_{ae}p_D}{\mu_{Aae}} & \frac{BR_{alb}p_D}{\mu_{Aalb}} \\ 0 & 0 & 0 & 0 \\ \frac{BR_{ae}p_D x_{ae}}{N_h\gamma} & \frac{BR_{ae}p_D x_{ae}}{N_h\gamma} & 0 & 0 \\ \frac{BR_{alb}p_D x_{alb}}{N_h\gamma} & \frac{BR_{alb}p_D x_{alb}}{N_h\gamma} & 0 & 0 \end{bmatrix}$$

Basic Reproductive Number (R_0)

The basic reproductive number is the spectral radius (dominant eigenvalue) of the next-generation matrix:

$$R_0 = p_D\sqrt{\frac{BR_{ae}^2\mu_{Aalb}x_{ae} + BR_{alb}^2\mu_{Aae}x_{alb}}{N_h\gamma\mu_{Aae}\mu_{Aalb}}}$$

Note: matrix algebra was calculated with Python.
